# Synergistic Interactions of a Synthetic Lubricin-Mimetic with Fibronectin for Enhanced Wear Protection

**DOI:** 10.3389/fbioe.2017.00036

**Published:** 2017-06-28

**Authors:** Roberto C. Andresen Eguiluz, Sierra G. Cook, Mingchee Tan, Cory N. Brown, Noah J. Pacifici, Mihir S. Samak, Lawrence J. Bonassar, David Putnam, Delphine Gourdon

**Affiliations:** ^1^Department of Materials Science and Engineering, Cornell University, Ithaca, NY, United States; ^2^Meinig School of Biomedical Engineering, Cornell University, Ithaca, NY, United States; ^3^Department of Physics, University of Ottawa, Ottawa, ON, Canada

**Keywords:** lubricin-mimetic, fibronectin, wear protection, bottlebrush polymer, surface forces apparatus

## Abstract

Lubricin (LUB), a major mucinous glycoprotein of mammalian synovial fluids, is believed to provide excellent lubrication to cartilage surfaces. Consequently, when joint disease or replacement leads to increased friction and surface damage in the joint, robust synthetic LUB alternatives that could be used therapeutically to improve lubrication and surface protection are needed. Here, we report the characterization of a lubricating multiblock bottlebrush polymer whose architecture was inspired by LUB, and we investigate the role of fibronectin (FN), a glycoprotein found in the superficial zone of cartilage, in mediating the tribological properties of the polymer upon shear between mica surfaces. Our surface forces apparatus (SFA) normal force measurements indicate that the lubricin-mimetic (mimLUB) could be kept anchored between mica surfaces, even under high contact pressures, when an intermediate layer of FN was present. Additional SFA friction measurements show that FN would also extend the wearless friction regime of the polymer up to pressures of 3.4 MPa while ensuring stable friction coefficients (μ ≈ 0.28). These results demonstrate synergistic interactions between mimLUB and FN in assisting the lubrication and wear protection of ideal (mica) substrates upon shear. Collectively, these findings suggest that our proposed mimLUB might be a promising alternative to LUB, as similar mechanisms could potentially facilitate the interaction between the polymer and cartilage surfaces in articular joints and prosthetic implants *in vivo*.

## Introduction

Successful biomimetic lubricants should prevent wear and reduce friction between contacting surfaces when subjected to (i) high loading pressures, (ii) a wide range of sliding speeds, and (iii) large shearing distances (with respect to the contact area between surfaces): conditions that are all found in synovial joints. Another notable characteristic of synovial joints is their ability to rapidly switch between biolubrication modes, which include boundary and elastohydrodynamic lubrication mechanisms (Swann et al., 1974; Roberts et al., 1982; Jahn et al., 2016). Nature has developed a solution to overcome fast changing sliding speeds with efficient lubrication, anti-adhesion, and robust wear protection: a mucinous glycoprotein known as lubricin (LUB). It is found in mammalian synovial fluids (Radin et al., 1970; Swann et al., 1981) and is reported to be a key contributor to the exceptional tribological properties of synovial joints, not only in reducing the friction between cartilage surfaces but also in protecting them against potential wear during shear (Rhee et al., 2005). Previous work performed on LUB physisorbed onto mica using the surface force apparatus (SFA, same technique as used in this report) reported very low friction coefficients (μ = 0.02−0.04) when sheared below 0.5 MPa contact pressures, increasing to μ = 0.2−0.6 at higher pressures (Zappone et al., 2007). This remarkable lubrication is believed to arise from the bottlebrush structure of LUB combined with its ability to self-associate into dimers or multimers that anchor robustly to the cartilage surface (Swann et al., 1985). LUB has been reported to bind to cartilage through its carboxyl-terminus (Jones et al., 2007), building a brush-like layer of dimers forming an arc-like (loop) architecture (Zappone et al., 2007, 2008; Andresen Eguiluz et al., 2015). Importantly, LUB is able to bind to various extracellular matrix components, including collagen (COL) (Chang et al., 2013), hyaluronan (HA) (Das et al., 2013), and fibronectin (FN) (Elsaid et al., 2007), a prominent glycoprotein which, in synovial joints, is only found in the superficial zone of cartilage (Balazs, 2009), i.e., at the interface between cartilage and synovial fluid. It has also been suggested that, among all aforementioned components, LUB’s highest affinity is for FN (Elsaid et al., 2007).

Inspired by natural lubricants with bottlebrush architecture, various mimetic analogs have been explored (McCutchen, 1968; Yan et al., 2004; Iruthayaraj et al., 2008; Pettersson et al., 2008; Perry et al., 2009; Krivorotova et al., 2010; Banquy et al., 2014a; Lawrence et al., 2015; Samaroo et al., 2016). Overall, these studies suggest that bottlebrush architecture provides efficient lubrication in aqueous environments because it prevents interdigitation between brushes anchored to opposing surfaces. Both the steric repulsion between protruding molecular chains and the presence of a hydration layer likely contribute to the swelling and stretching of the brushes, which then provide low friction. However, the inability of brushes to efficiently lubricate junctions under high pressures (Lee et al., 2003; Benz et al., 2004) suggests that hydration itself is insufficient to guarantee decent lubrication and that the polymers must also be strongly anchored to the surfaces, either *via* their central backbone (Yan et al., 2004; Pettersson et al., 2008) or *via* their terminal moieties (Banquy et al., 2014a). Strong anchoring can be achieved by covalent bonding (Yan et al., 2004) or by electrostatic interactions (Huang et al., 2001; Banquy et al., 2014a). In the particular case of bottlebrush copolymers decorated with poly(ethylene glycol) (PEG) side chains, both grafting density (Perry et al., 2009) and length of side chains (Pettersson et al., 2008) in the interfacial regions were also shown to be key mediators of lubrication, and friction coefficients as low as μ = 0.06 were reported (Lee et al., 2003). Yet, despite these low friction coefficients, PEG-based brushes have not been commonly utilized as water-based lubricants but rather as protective and/or anti-fouling coatings (Lee et al., 1990; Heuberger et al., 2005; Fan et al., 2006).

While the interactions of LUB with COL (Chang et al., 2013) and HA (Chang et al., 2008; Greene et al., 2011; Das et al., 2013) have been widely investigated, the role of FN in enhancing LUB-mediated low friction and wear protection at the superficial zone of cartilage has only recently been proposed (Andresen Eguiluz et al., 2015). In this study, we report the characterization of a LUB-mimetic bottlebrush polymer, named mimLUB, through the combination of atomic force microscopy (AFM) and SFA. This combination allowed us to assess both structural and tribological (adhesion, friction, and wear) properties of the polymer when sheared between model mica surfaces, in presence or absence of FN. MimLUB consists of a long and flexible poly(acrylic acid) (pAA) backbone grafted with PEG side chains (Samaroo et al., 2016, 2017). It possesses a thiol terminus on one end to anchor it to FN while the other end is not functionalized. Our choice of pAA and PEG polymers is predominantly based on the excellent biocompatibility reported for those two polymers (Yim et al., 2009). To assess mimLUB tribological properties at the molecular scale and determine the role of FN in mediating these properties, we sheared mimLUB between either bare or FN-coated mica surfaces. Our data indicate that the presence of FN prevents mimLUB from being squeezed out of the shearing junction, which significantly extends its wearless friction regime (up to physiologically relevant pressures), while maintaining friction coefficients similar to those measured across native LUB under identical experimental conditions (Andresen Eguiluz et al., 2015). Collectively, these findings suggest that, when combined with FN, our proposed lubricin-mimetic (mimLUB) provides enhanced wear protection. Finally, because LUB is a complex molecule that would require onerous synthesis due to the large number of amino acid repeats in the protein core and the high degree of glycosylation (Jay, 2004; Jones et al., 2007; Flannery et al., 2009), mimLUB might be a promising clinical alternative over recombinant LUB to treat unhealthy articular joints and prosthetic implants *in vivo*.

## Materials and Methods

### Synthesis of mimLUB

Acrylic acid (AA, 99.5%) stabilized with 200 ppm 4-methoxyphenol, methanol (99.8%), and sodium borate buffer were obtained from VWR (Radnor, PA, USA). 4,4′-Azobis-(4-cyanopentanoic acid) (A-CPA) and 4-cyano-4-(phenylcarbonothioylthio)pentanoic acid (CPA-DB) (>97% HPLC) was obtained from Sigma-Aldrich (St. Louis, MO, USA). Methoxy-poly(ethylene glycol)-amine powder (PEG-NH_2_) was obtained from Jenkem Technologies (Beijing, China) and 4-(4,6-dimethoxy-1,3,5-triazin-2-yl)-4-methylmorpholinium chloride (DMTMM) was obtained from TCI America (Portland, OR, USA). All chemicals were used as received unless otherwise specified.

### Synthesis and Characterization of pAA Backbone

#### Synthesis

Poly(acrylic acid) was synthesized by RAFT polymerization using AA, A-CPA as initiator (I) and CPA-DB as chain transfer agent (CTA) under anhydrous, airtight, and dark conditions in methanol. AA concentration was maintained at ~3.8 mM, while [AA]:[I]:[CTA] was 762:0.25:1. The general reaction scheme is as follows: AA was added to a flame-dried 5 ml brown ampule with one magnetic stir bar, to which CPA-DB dissolved in 2.9 ml of nitrogen-purged methanol was added, followed by A-CPA dissolved in 0.7 ml of nitrogen-purged methanol. Nitrogen gas was bubbled through the reaction mixture after addition of each reagent for several minutes to prevent oxygen gas influx. After the last nitrogen purge, the reaction ampule was flame sealed, placed in a 60°C oil bath to initiate polymerization, and allowed to stir for 48 h. Upon reaction completion, the ampule neck was broken to expose the reactants to air and the reaction was cooled in ice to stop the polymerization. The solution was diluted with water, dialyzed against deionized water for 3 days, and then lyophilized to obtain a white, waxy powder.

#### Characterization

Poly(acrylic acid) was dissolved in D_2_O and characterized using ^1^H NMR (INOVA 400 MHz). The methylene and methine chemical shifts are at 1.5–2 and 2.25–2.75 ppm, respectively. Molecular weight was determined by Waters gel permeation chromatography (GPC) system (Waters 1515 Isocratic HPLC Pump, Waters 2414 Refractive Index Detector) using poly(methacrylic acid) standards and phosphate-buffered saline (PBS) (pH 7.4) as the mobile phase at 30°C.

### Synthesis of the pAA-*graft*-PEG (pAA-*g*-PEG) Bottlebrush Polymer

The pAA-*g*-PEG copolymer was synthesized by polymer analogous conjugation of monoamine-functionalized PEG to the pAA backbone using DMTMM as the coupling agent. pAA was dissolved in 0.1 M borate buffer (pH 8.5) at 3.3 mg/ml, with reactant mole ratios of [AA]:[DMTMM]:[PEG] set at 1:2:2. The general reaction is as follows: pAA (*M*_w_ 60,000) and *M*_n_ PEG-amine (*M*_w_ 2,000) were dissolved in 3 ml borate buffer in a 10 ml flask with magnetic stir bar. DMTMM was dissolved in 0.6 ml borate buffer and added dropwise into the reaction flask with the final pH adjusted to 6–7 using 1 M HCl. The conjugation reaction was conducted for 24 h at room temperature, dialyzed against deionized water for 3 days, and lyophilized to obtain a white powder. The tail end of pAA has a thiolcarbionylthio group that is cleaved during the PEG conjugation step exposing a free thiol group. The assigned nomenclature for the polymer brushes are given as pAA(*a*)-*g*-PEG(*b*), where *a* and *b* are molecular weights of pAA and PEG, respectively, and *g* is the grafting ratio defined by the moles of PEG over the moles of AA monomers in the pAA backbone used during the reaction. pAA(60)-2-PEG(2) and PEG calibrations standards were sent to the Biophysics Resource of Keck Laboratory at the Yale School of Medicine to be analyzed by the DAWN Helios multi-angle laser light scattering size exclusion chromatography system (MALLS/SEC). A Superose 6 column was used to fractionate the samples at ambient temperatures. pAA(60)-2-PEG(2) was dissolved at 3 mg/ml in PBS solution containing 120 mM NaCl, 10 mM phosphate salt, and 2.7 mM KCl (pH 7.4), and sonicated for 15 min before injection into the SEC using a dn/dc value of 0.135 ml/g. COOH groups on pAA are the potential conjugation sites for PEG. With this premise, the percent conjugation of PEG onto pAA was calculated from the molecular weight (*M*_w_) of pAA(60)-2-PEG(2). This specific mimLUB architecture has shown the lowest friction coefficient on articular cartilage from a library of eight polymers (Samaroo et al., 2016).

### Preparation of mimLub and FN Solutions

The synthesis of our LUB-inspired synthetic polymer mimLUB has been recently described in detail by our collaborators (Samaroo et al., 2016). The polymer, with average molecular weight of 1,400 kDa, was dissolved in PBS (PBS from EMD, Billerica, MA, USA) with a final concentration of 3 mg/ml. The solution was sonicated for 30 min using 18 MΩ Milli-Q water (Millipore Corporation, Billerica, MA, USA) to completely dissolve the mimLUB. A FN solution of 1 mg/ml in PBS was purchased from Sigma-Aldrich (St. Louis, MO, USA). Low concentration aliquots of human plasma FN at 0.3 mg/ml in PBS were prepared and stored at −80°C, and thawed when needed. All glassware used in the preparation was cleaned with ethanol and rinsed with DI.

### Atomic Force Microscopy

Atomic force microscopy measurements were performed in air using a commercial AFM (MFP-3D, Asylum, Sta. Barbara, CA, USA) to assess the nanostructure of mimLUB adsorbed onto mica. Conical SiO_2_ probes with nominal radius of curvature of 9 nm mounted on compliant (*k* = 42 N/m) levers (AC160TS, Olympus, USA) or triangular pyramid Si tips with *k* = 93 N/m lever and 10 nm radius of curvature from Appnano (model ACCESS-NC-A) were used for AC mode imaging. Images were taken over a range of 2 µm × 2 µm, at a frequency of 1 Hz and 1,536 × 1,536 pixels for maximal resolution. Image analysis was performed in Gwyddion (Czech Metrology Institute) and ImageJ (NIH). AFM samples consisted of freshly cleaved mica substrates, (i) spin-coated at 2,000 rpm for 1 min with 100 µl of a dilute mimLUB solution in DI water (0.3 mg/ml) and left 1 h for complete drying, or (ii) incubated for 30 min, rinsed with DI water, and left 1 h for complete drying.

### Surface Forces Apparatus

Normal and friction forces between two mimLUB-coated mica surfaces were measured using the SFA Mark III (SurForce, LLC, Sta. Barbara, CA, USA) using well-established procedures (Gourdon et al., 2004). Briefly, two freshly cleaved back-silvered mica sections (S&J Trading, Glen Oaks, NY, USA) were glued onto semi-cylindrical silica disks (*R* ≈ 1 cm) with UV curing glue (Norland 61, Cranbury, NJ, USA). The disks were mounted in a cross-cylindrical configuration and the absolute separation distance between them, *D*, was measured in real time by multiple beam interferometry (MBI). Additionally, MBI was used to monitor the onset of wear of the shearing surfaces: both shape and intensity of interference fringes were used as indicators of shape (and size) of the contacting junction and presence of shear-induced wear debris (Gourdon and Israelachvili, 2003; Banquy et al., 2014b; Andresen Eguiluz et al., 2015). Before functionalizing the mica surfaces with either mimLUB or FN + mimLUB, mica–mica contact in air was measured to determine the reference distance, *D* = 0. To quantify normal forces, the lower surface was mounted onto a compliant horizontal double cantilever spring ($k_{⊥}=\text{590 N/m}$) and displaced at a constant approach speed of circa 5 nm/s. For tribological characterization requiring higher applied pressures, the lower surface was mounted onto a stiffer horizontal spring ($k_{⊥}=\text{1,650 N/m}$), whereas the upper surface was mounted onto a vertical double cantilever spring ($k_{∥}=\text{700 N/m}$) holding strain gages to measure friction forces. Shearing was achieved *via* a ceramic bimorph slider, and shearing velocities of *V* ≈ 0.3, 3, and 30 µm/s were used in our experiments, corresponding to shearing frequencies of 0.005, 0.05, and 0.5 Hz, which is the range of frequencies experienced during physical activity such as walking (Balazs, 2009). MBI fringes of equal chromatic order were collected using a SP2300 photospectrometer (Princeton Instruments, NJ, USA) with a 600 g/mm grating and 500 nm blaze, digitalized with a ProEM CCD camera (Princeton Instruments, NJ, USA), and visualized using Lightfield v4.0 (Princeton Instruments, NJ, USA). Friction forces were acquired and quantified with a NI USB-6210 and LabView v8.6 (National Instruments, Austin, TX, USA), respectively.

### Surface Functionalization with mimLUB

Two protocols were carried out for surface functionalization: mimLUB was adsorbed either (i) directly onto bare mica or (ii) onto mica previously coated with FN. For protocol (i), freshly prepared mica surfaces were incubated with 50 µl mimLUB solutions at 3 mg/ml in PBS for 1 h and rinsed with PBS. For protocol (ii), freshly prepared mica surfaces were first incubated with 50 µl of FN solution (0.3 mg/ml in PBS) for 1 h and rinsed with PBS. 50 µl of bovine serum albumin at 0.02 mg/ml in PBS were then added for 30 min to block non-specific interactions and rinsed with PBS. Finally, these FN-anchored mica surfaces were incubated with 50 µl mLUB solutions at 3 mg/ml in PBS for 1 h and rinsed with PBS. In addition, we used two different experimental conditions: surfaces were sheared either in PBS or in mimLUB (3 mg/ml) solution. All surface functionalization steps were carried out by injecting liquid droplets between mica surfaces that were previously mounted in the SFA chamber to ensure similar protein adsorption on both upper and lower surfaces. All steps were performed in a laminar flow cabinet to prevent particle contamination.

### Statistical Analysis

**Figure 1:**
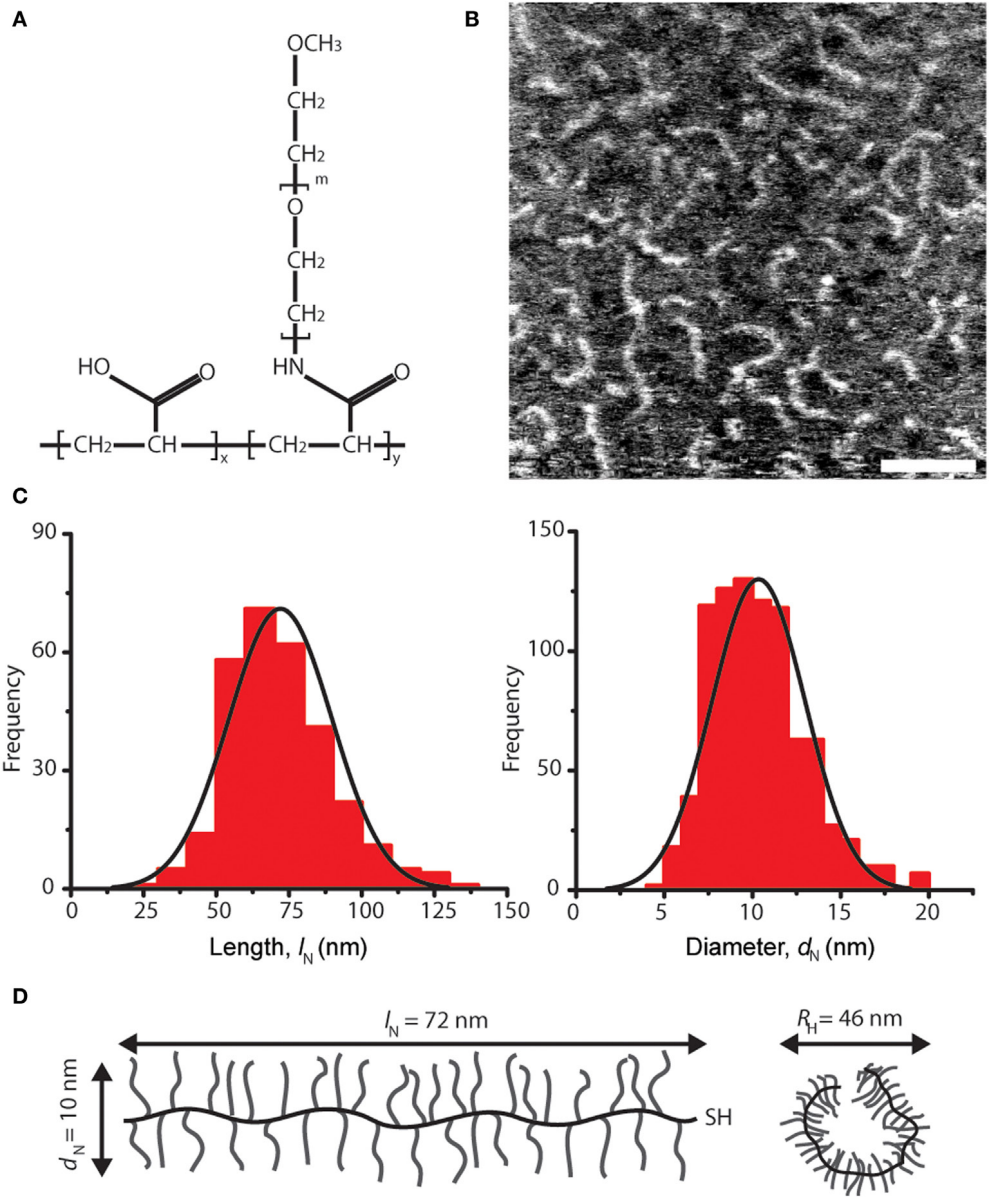
Architecture and dimensions of lubricin-mimetic (mimLUB). **(A)** Structure of the pAA-*graft*-PEG bottlebrush polymer mimLUB. In our study, *x* ≈ 185, *y* ≈ 650, *m* ≈ 45, and *M*_W_ ≈ 1,400 kDa. **(B)** Atomic force microscopy height micrograph of mimLUB chains spin-coated onto freshly cleaved mica, scale bar = 50 nm. **(C)** Number-average contour length *l*_N_ and number-average molecular diameter *d*_N_. Images = 6, molecules = 875. **(D)** Schematic representations of mimLUB with *l*_N_, *d*_N_, and *D*_H_.

**Figure 2:**
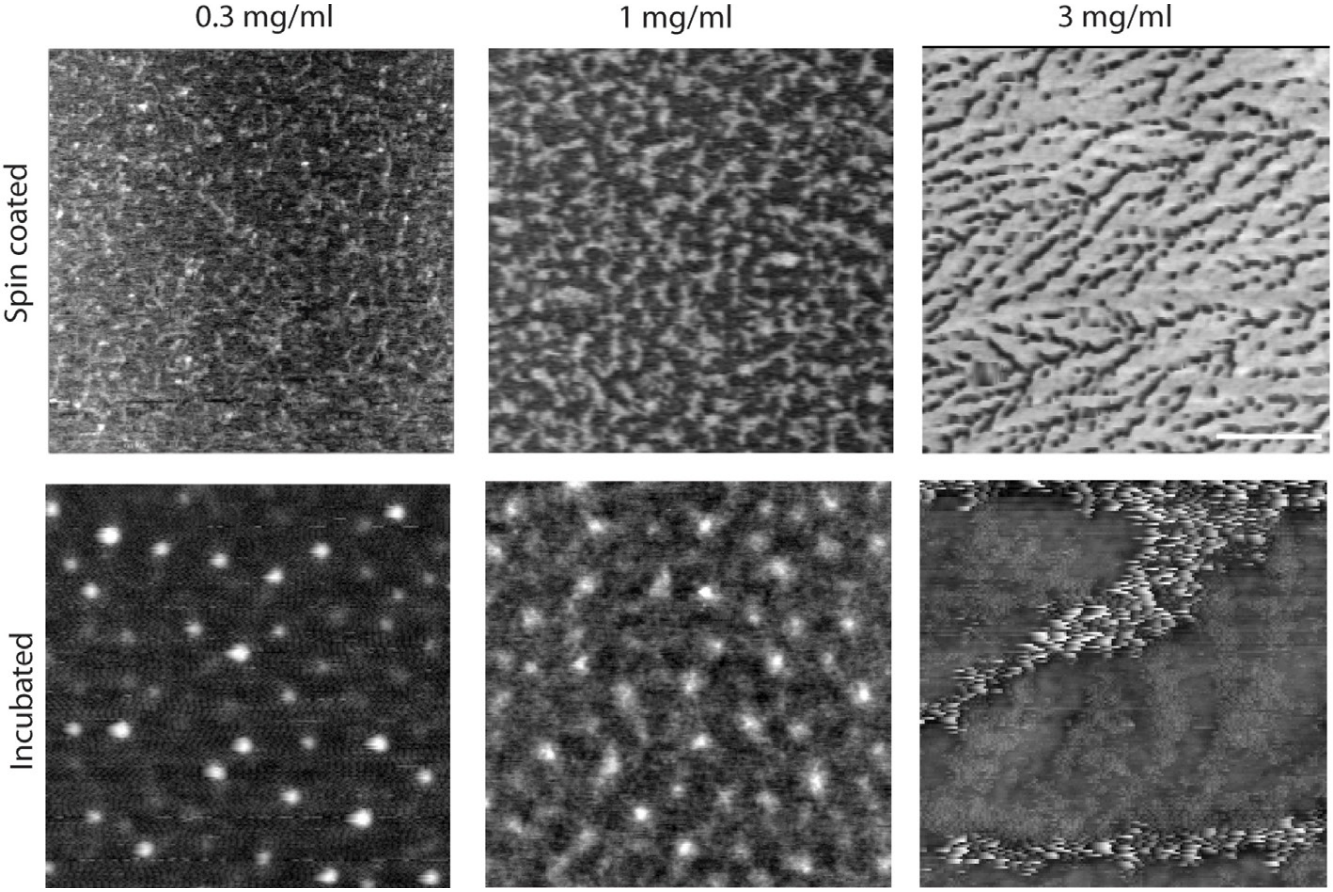
Effect of concentration and incubation protocol on mimLUB network formation (top panels). Atomic force microscopy height micrograph of spin-coated mimLUB indicates the presence of a polymeric network at all concentrations, with coverage that increases with increasing concentration, suggesting that mimLUB molecules agglomerate and entangle (bottom panels). Incubated samples form polymer agglomerates of varied dimensions. Scale bar = 500 nm.

**Figure 3:**
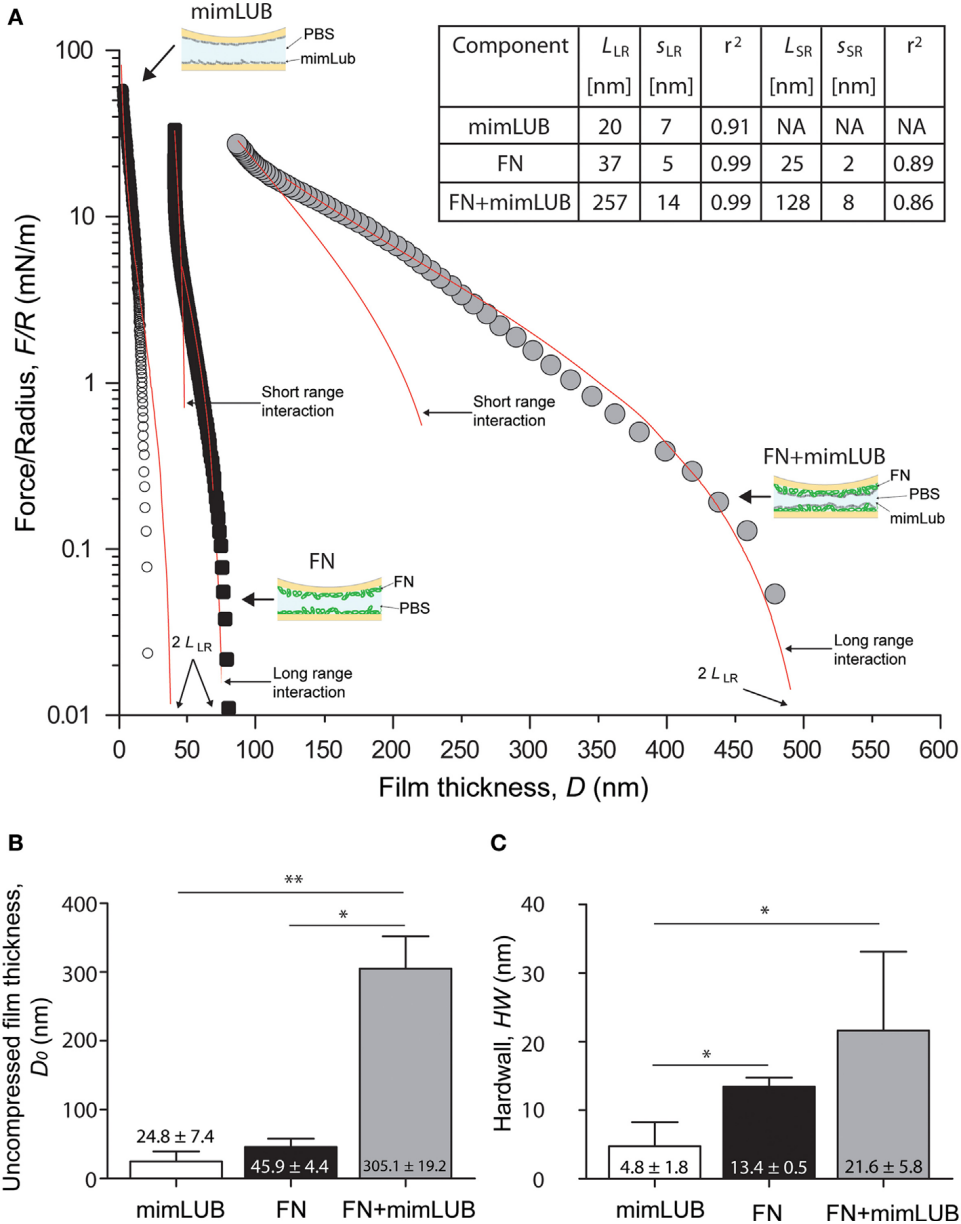
Role of fibronectin (FN) in normal interactions between mimLUB-coated surfaces. **(A)** Normal force $F_{⊥}$ normalized by the surface radius of curvature *R* between two mica surfaces coated with a mimLUB layer (white circles), coated with a FN layer (black squares), and coated with a FN + mimLUB layer (gray circles) as a function of total film thickness, *D*. Forces are measured upon approach at a constant velocity of 5 nm/s. **(B)** Bar charts of average film thicknesses at rest, *D*_0_, and **(C)** average “hardwall” thicknesses, HW for mimLUB (black), FN (gray), and FN + mimLUB (white) films. Values reported as mean + SD of one-way ANOVA tests. In all cases, *p* < 0.05 is indicated by a single star and *p* < 0.01 by two stars. All data were fitted using the AdG model (Eq. 1).

**Figure 4:**
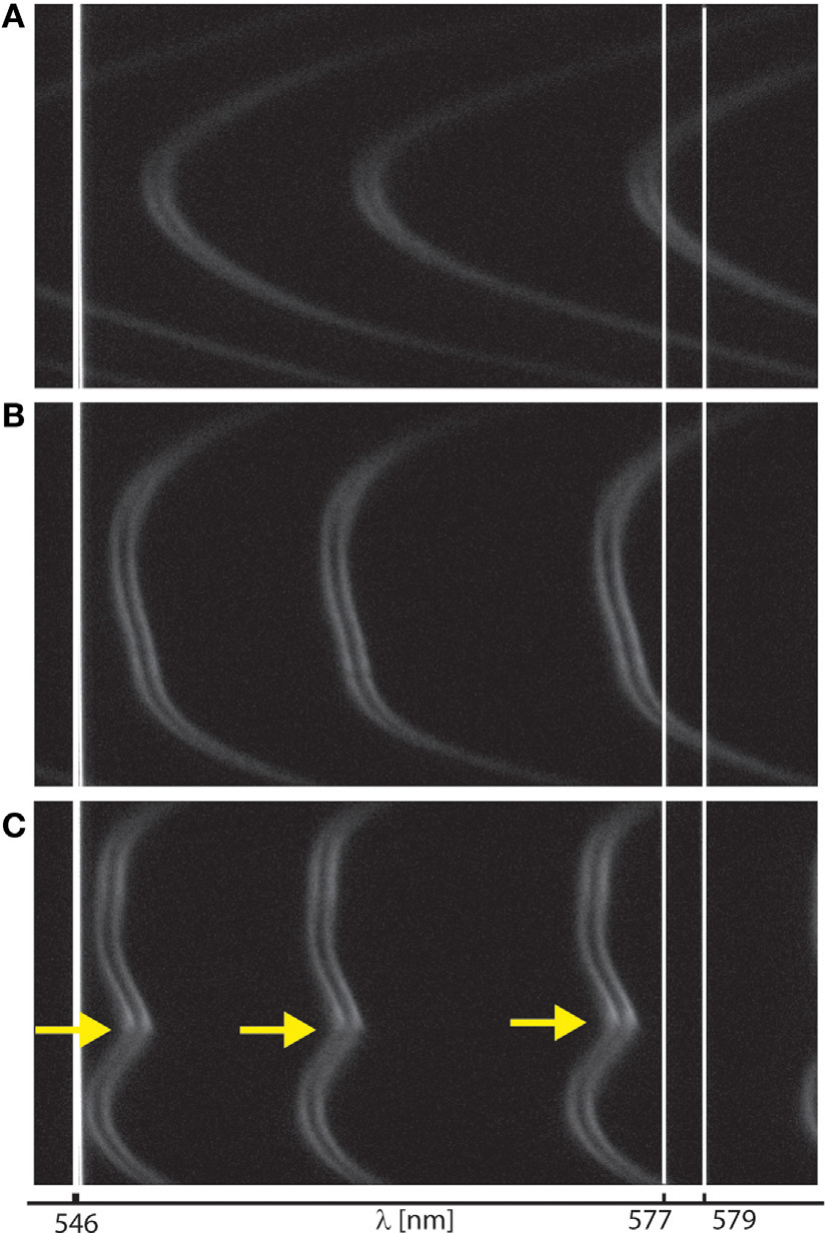
Interferometry for monitoring surface wear during shear. Representative interference fringes recorded during surface forces apparatus shearing measurements, as visualized **(A)** at onset of interaction, **(B)** at large normal loads deforming contact junction, and **(C)** at onset of wear, indicated by yellow arrows. Fringes shape and shift (relative to initial mica–mica contact without film, not shown here) allowed us to monitor surface wear as well as film thickness and size of wear debris.

**Figure 5:**
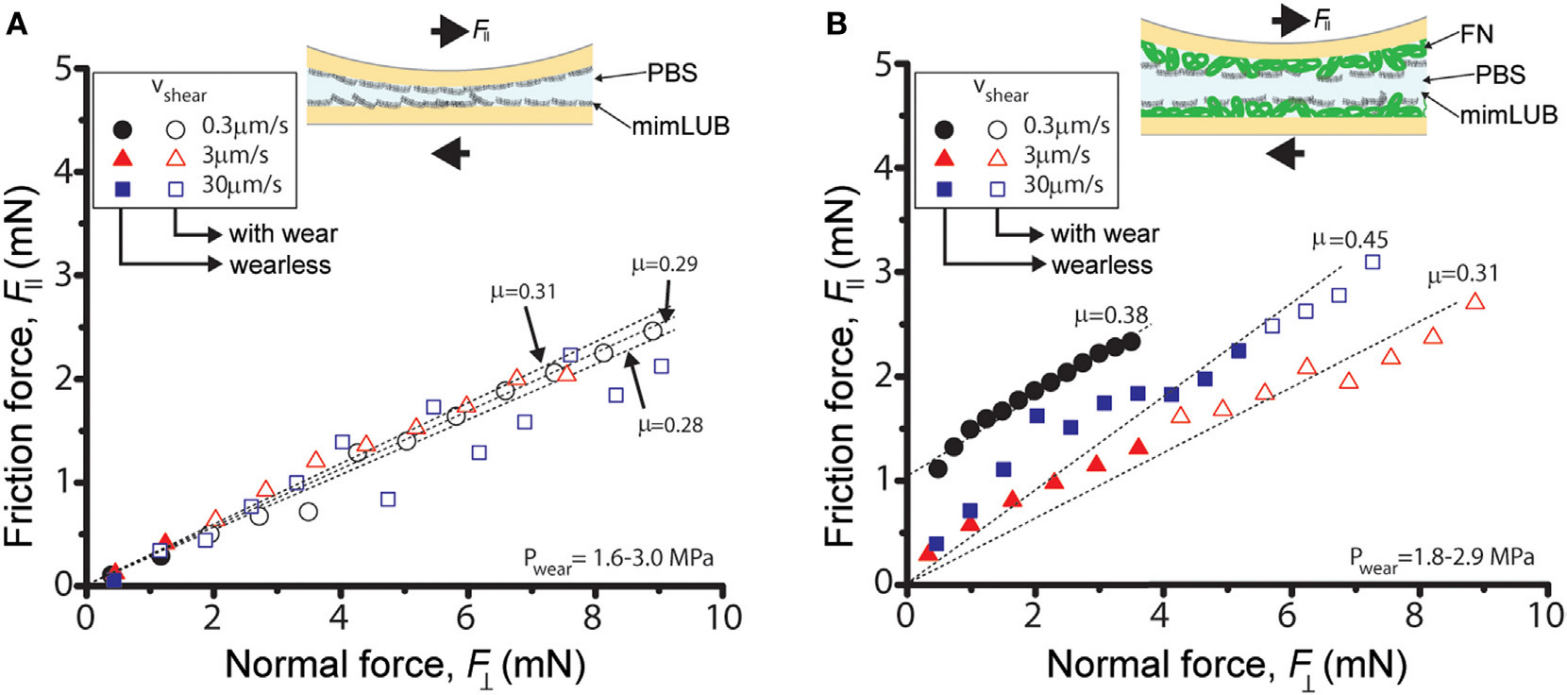
Role of fibronectin (FN) in friction and wear of sheared mimLUB-coated surfaces across phosphate-buffered saline (PBS). Friction force $F_{∥}$ as a function of normal force $F_{⊥}$ measured across PBS between **(A)** mica surfaces incubated with mimLUB and **(B)** FN-coated mica surfaces incubated with mimLUB. The surfaces were sheared in PBS at sliding velocities of *V* = 0.3 µm/s (black circles), *V* = 3 µm/s (red triangles), and *V* = 30 µm/s (blue squares). Open symbols indicate measurements after the occurrence of wear in the sheared junction.

**Figure 6:**
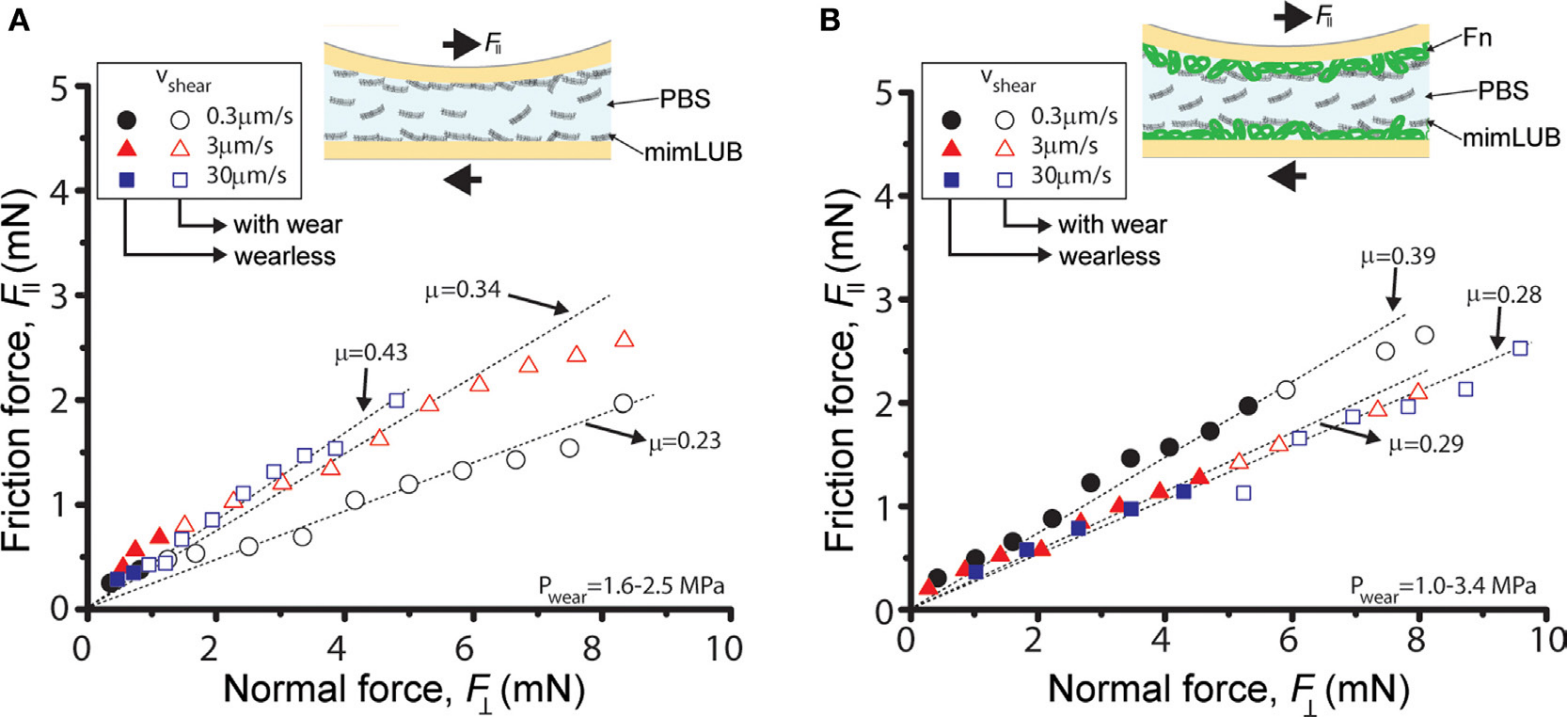
Role of fibronectin (FN) in friction and wear of sheared mimLUB-coated surfaces across a mimLUB solution. Friction force $F_{∥}$ as a function of normal force $F_{⊥}$ measured across mimLUB solution (3 mg/ml) between **(A)** mica surfaces coated with mimLUB and **(B)** mica surfaces coated with FN + mimLUB. The surfaces were sheared at sliding velocities of *V* = 0.3 µm/s (black circles), *V* = 3 µm/s (red triangles), and *V* = 30 µm/s (blue squares). Open symbols indicate measurements after the surfaces became wear (with wear).

All tribological data and images displayed in the figures throughout the manuscript are representative of two independent SFA experiments. Frequency histograms in Figure 1C display 875 individual measurements from six images acquired through two AFM experiments. Figure 2 shows representative AFM height micrographs. Figure 3 shows representative profiles of six measurements performed on two different positions from two independent experiments. Bar-plots represent mean ± SD. One-way ANOVA tests were conducted using Graph Pad Prism 5 to compare the means between all three experimental conditions. Statistical significance was considered when *p* < 0.05 at the 95% confidence level. Figures 4–6 show representative MBI patterns and friction force $F_{∥}$ as a function of normal force $F_{⊥}$ curves from two independent experiments.

## Results

### Molecular Characterization of mimLUB

We characterized a pAA-*g*-PEG-based polymer, named mimLUB, whose design is depicted in Figure 1A. It possesses a long region with bottlebrush architecture composed of a flexible pAA backbone decorated with PEG side chains. To determine the dimensions of single molecules, we spin coated freshly cleaved mica surfaces with low concentrations of mimLUB solutions (0.3 mg/ml in deionized water) and imaged individual polymer chains in air (intermittent contact) using AFM. As shown in Figure 1B, the molecules exhibited worm-like morphology; their average contour length *l*_N_ and average diameter *d*_N_ were 72.0 ± 17.8 and 10.3 ± 2.6 nm, respectively (frequency histograms for *l*_N_ and *d*_N_ are displayed in Figure 1C). The hydrodynamic diameter, *D*_H_, quantified in solution *via* dynamic light scattering, was *D*_H_ = 46.0 nm. All molecular dimensions are summarized in Figure 1D. Additionally, the percent conjugation calculated from the molecular weight (*M*_W_) was 83%, the measured polydispersity index obtained from GPC-MALLS was *M*_w_/*M*_n_ = 1.3, and *M*_w_ was 1,400 kDa.

We next investigated the effects of mimLUB concentration and incubation conditions on the resulting surface coverage and distribution. Three different concentrations (0.3, 1.0, and 3 mg/ml) and two different incubation protocols were tested: (i) spin coating of mimLUB solutions (2,000 rpm) or (ii) incubation of mimLUB solutions for 30 min followed by thorough rinsing with deionized water, both onto freshly cleaved mica substrates. Our AFM imaging indicates that all spin-coated conditions (Figure 2, top panels) resulted in a uniform network of densely packed mimLUB molecules, with substrate coverage increasing with increasing concentration. In contrast, incubated mimLUB samples (Figure 2, bottom panels) displayed uniformly distributed agglomerates of varied height, with distance between aggregates decreasing with increasing concentration. Overall, these results show that mimLUB molecules self-aggregate and form an interconnected polymeric network rather than evenly distribute into a single layer across the surfaces.

### Role of FN in Mediating Normal Interactions between mimLUB-Coated Mica Surfaces

To determine whether the presence of FN affects the interactions between mimLUB brushes, we first used the SFA to perform normal (compressive) measurements of mimLUB-coated mica surfaces in presence or absence of an underlying FN layer in PBS at 25°C. Figure 3A shows the normal interaction forces, reported as $F_{⊥}/R$, $F_{⊥}$ being the normal force and *R* the surface radius of curvature, between (i) mimLUB films directly adsorbed onto mica surfaces and (ii) mimLUB films adsorbed onto mica previously coated with FN. Interactions measured across FN films alone are also displayed for comparison. Data were fitted with the Alexandre-de Gennes model (AdG), which usually describes the normal interactions between surfaces holding neutral polymer brushes (Israelachvili, 2011):

(1) F⊥(D)R=16πkTL35s3[7(2LD)54+5(D2L)74−12]

where *k* is the Boltzmann constant, *T* is temperature, and *L* and *s* are the relaxed brush length and average grafting spacing, respectively, used as fitting parameters for the model. The use of the AdG model to describe the behavior of charged mimLUB chains is justified by the high salinity of the surrounding medium (PBS, 150 mM). Although mimLUB is a polyelectrolyte, the numerous counterions in PBS are expected to screen most of the electrostatic interactions between chains so that mimLUB can be treated as a neutral bottlebrush polymer. We identified both long-ranged (LR) and short-ranged (SR) brush regimes in our mimLUB + FN data, similar to what has been observed for LUB + FN (Andresen Eguiluz et al., 2015). Importantly, by using the AdG model to describe FN (alone), we do not intend to imply that FN layers also possess a well-defined brush structure. Rather, the close agreement between our data and the AdG theory suggests that FN films adsorbed onto mica in PBS can be described as a repulsive “brush-like” layer. In all our measurements, the interactions were reversible: normal forces were purely repulsive, namely, no measurable adhesion and no hysteresis between approach and retraction were observed (data not shown). In absence of FN (mimLUB), a single regime was detected with interaction forces starting at ≈40 nm (corresponding to 2*L*_LR_). In contrast, two regimes were observed in presence of FN (FN + mimLUB), with long-range interactions starting at ≈500 nm (2*L*_LR_) followed by short-range interactions at ≈250 nm (2*L*_SR_). The existence of two regimes could be explained by a change in conformation of mimLUB, transitioning from a disordered structure (possibly loosely assembled agglomerates) to a more ordered single layer of horizontally oriented mimLUB molecules. As contact pressure increases, any mimLUB that was not directly anchored to the FN layer was squeezed out from the contacting junction. These results are summarized in Figure 3A. Average values of onset of interactions, i.e., uncompressed film thicknesses (*D*_0_), are displayed in Figure 3B and indicate that FN + mimLUB films were significantly thicker not only than mimLUB or FN films alone but also than the sum of both of them. At high pressures, the mimLUB film thickness was close to zero, indicating that (almost) no mimLUB molecules remained anchored to mica. Average “hardwall” values (HW), indicative of film thicknesses under maximum applied load, are shown in Figure 3C and confirm that FN + mimLUB films were thicker than the mimLUB films alone, even under high load. Furthermore, the sum of the HW values of FN alone added to the diameter of mimLUB equaled the HW value of FN + mimLUB film, suggesting that upon compression, mimLUB molecules were lying flat against the FN layer. Collectively, our results indicate that FN-mimLUB interactions are strong enough to retain a layer of mimLUB anchored to the mica surface and prevent it from being expelled from the junction, even when it is subjected to large compressive forces.

### Role of FN in Mediating Lubrication between mimLUB-Coated Mica Surfaces

We next investigated the role of FN in the lubrication and wear protection of mimLUB-coated surfaces upon shearing. The SFA was, therefore, used to apply shear and measure both the friction forces and the onset of wear between mimLUB-coated mica surfaces, in presence or absence of underlying FN. All systems were sheared either in PBS or in dilute mimLUB medium, at 25°C. Figure 4 summarizes the evolution of the interference fringes recorded over a full shearing experiment, from onset of surface interaction (Figure 4A) followed by deformation (flattening) of the surfaces upon increasing load (Figure 4B), as indicated by a flattening of the tip of the fringes, ending in local surface wear when no intermediate FN layer was present (yellow arrow in Figure 4C). At high loads, the contact area, *A*, was directly measured from the fringes flat region, and the average pressure across the compressed films was calculated as:

(2) $$P=\frac{F_{\text{High}}}{A}$$

where *F*_High_ is the normal force at high loads, and *A* is the contact area of the compressed junction. At low load, as the surfaces did not clearly deform (Figure 4A), contact pressures, *P*(*F*_Low_), were evaluated from Gaisinskaya-Kipnis and Klein (2016):

(3) P(FLow)=P(FHigh)×(FLowFHigh)13

where *F*_Low_ is the normal force at low loads.

Figure 5 displays the friction force $F_{∥}$ as a function of normal force $F_{⊥}$ measured between mimLUB-coated surfaces sheared across PBS, in presence or absence of FN coating. Without FN (Figure 5A), wear between surfaces occurred almost immediately after shear started, as indicated by open symbols in the friction data. Wear was assessed through the irreversible deformation and non-continuous intensity of consecutive (odd and even) interference fringes (as shown in Figure 4C), which further suggests that both the mimLUB film and the underlying mica were damaged simultaneously (Andresen Eguiluz et al., 2015). The resulting wear-associated friction forces $F_{∥}$ were nearly independent of shearing velocities and proportional to applied normal load, vanishing at $F_{⊥}=0$. The average friction coefficient, defined as $\mu =\Delta F_{∥}/\Delta F_{⊥},$ was equal to 0.290 ± 0.015. Wear debris were noticed at extremely low loads ($F_{⊥}<1.5\text{ mN}$, equivalent to *P* = 3.0 and 1.7 MPa), at low and intermediate speeds, but not at high speeds. The high pressures occurring at low loads are due to the small contact areas encountered during the loading steps.

In contrast, the (higher) friction forces measured in the presence of FN (Figure 5B) were sensitive to shearing velocity, did not depend linearly on the applied load $F_{⊥}$, and did not vanish at $F_{⊥}=0$, particularly at the lowest sliding speed (0.3 µm/s). Interestingly, wearless friction was sustainable up to circa 4 mN (for all speeds), and wear was triggered only at loads of 3.6 and 5.0 mN [corresponding to pressures of 3.0 and 1.8 MPa, i.e., within the physiological range (Morrell et al., 2005)] at speeds of *V* = 3.0 and 30.0 µm/s, respectively. However, at low shearing velocities, normal applied loads did not reach high enough values to induce wear. For comparison, the friction coefficient of FN alone was measured to be 0.223 ± 0.075; however, damage was reached at much lower shearing cycles (see all values reported in Table 1). Overall, our results indicate that FN significantly delays the onset of wear of FN + mimLUB sheared films, likely, by providing stronger anchorage of mimLUB molecules to the opposing surfaces, which permits shear-induced re-orientation of the film and limited interdigitation and/or squeeze out of molecules (as detailed in the Section “Discussion”).

**Table 1:** Summary of reported pressures.

Condition	*V* = 0.3 µm/s				*V* = 3.0 µm/s				*V* = 30.0 µm/s			
	*P*_onset_ (MPa)	*P*_max_ (MPa)	*P*_wear_ (MPa)	Cycles at *P*_wear_	*P*_onset_ (MPa)	*P*_max_ (MPa)	*P*_wear_ (MPa)	Cycles at *P*_wear_	*P*_onset_ (MPa)	*P*_max_ (MPa)	*P*_wear_ (MPa)	Cycles at *P*_wear_
mimLUB in phosphate-buffered saline (PBS)	1.8	5.1	3.0	15	1.0	2.9	1.7	21	1.2	1.6	3.2	54
mimLUB in mimLUB	1.1	3.1	1.6	15	1.8	4.5	2.6	29	1.3	2.3	1.9	90
Fibronectin (FN) + mimLUB in PBS	0.4	0.8			1.8	3.8	3.0	54	0.6	2.2	1.8	378
FN + mimLUB in mimLUB	1.4	3.8	3.4	57	0.9	2.8	2.4	71	0.6	1.4	1.1	198
FN			0.9	49			0.41	14			1.3	188
LUB^a^							0.4^c^					
FN + LUB^b^		4.0				4.5				14.0		

*Pressure at onset of interaction (*P*_onset_), maximum pressures reached at each condition (*P*_max_), and pressures at onset of wear (*P*_wear_) measured in our study. Values reported in literature for native LUB are shown for comparison. In parenthesis, shearing cycles*.

*^a^From Zappone et al. (2007)*.

*^b^From Andresen Eguiluz et al. (2015)*.

*^c^At 1.0 µm/s*.

To better mimic joint lubrication conditions, we next sheared the mimLUB-coated surfaces across a mimLUB solution (Figure 6). Here again, the tribological properties of the mimLUB, in particular its resistance to wear, were found to be improved by the presence of FN. All friction forces were proportional to applied normal loads and vanished at $F_{⊥}=0$ but, contrarily to what was observed in PBS, the friction coefficients depended on sliding speed. Figure 6A shows that, without FN, systematic surface wear occurred at extremely low loads ($F_{⊥}<1.5\text{ mN}$, equivalent to 1.6–2.6 MPa) regardless of sliding conditions. However, wear-associated friction coefficients increased with increasing shearing velocity. In contrast, the presence of FN drastically extended the wearless friction regime by postponing the formation of wear debris, which was triggered only at loads $F_{⊥}>4.5\text{ mN}$ (and large contact areas), i.e., under pressures above 3.4, 2.4, and 1.1 MPa (Figure 6B), for slow, intermediate, and fast shearing velocities, respectively. Overall, the presence of mimLUB in the shearing medium provided a modest enhancement of the wear protection of surfaces suggesting that surface-anchored mimLUB molecules (rather than free-floating mimLUB molecules in the medium) were likely responsible for enhanced lubrication and resistance to wear of the sheared surfaces. These results support the idea that robustly anchored mimLUB onto opposing surfaces are key to efficient lubrication and wear protection and confirm the likely role of FN in mediating these anchoring mechanisms.

## Discussion

This study aimed at characterizing the molecular structure and tribological performance of a mimLUB pAA-*g*-PEG copolymer (mimLUB) when anchored and sheared between model (mica) surfaces. Particular emphasis was placed on the role of FN (present in the superficial zone of cartilage) in facilitating the polymer’s ability to protect surfaces against wear during shear.

Overall, our main findings show that FN plays a key role in mediating the surface protection of mimLUB-coated surfaces during shear. Our normal force characterization combined with AdG theory (Figure 3) indicates that FN provides a more robust anchoring of mimLUB within the mica junction, as suggested by the larger short-range interaction distance measured for FN + mimLUB films than for FN alone. Additionally, the long-range interaction detected for FN + mimLUB occurs at larger distances than the sum of FN and mimLUB films interactions, suggesting that large mimLUB agglomerates may form before being squeezed out of the junction as load increases, leaving behind a single layer of mimLUB molecules well-anchored to the FN layer. This interpretation is further supported by the relatively large SD in our film thickness measurements. A possible anchoring mechanism could be attributed to the partially unfolded conformation (Andresen Eguiluz et al., 2015; Wang et al., 2015; Wu et al., 2015, 2017) FN adopts when adsorbed onto freshly cleaved mica surfaces, which would expose several cryptic cysteines (in particular, the ones located on FN type III_7_ and III_15_ modules) making them available to the deprotonated thiol moieties carried by mimLUB (Poole, 2015). Additionally, our friction data (Figures 5 and 6) indicate that FN and mimLUB act synergistically to extend wearless friction up to higher pressures and/or higher number of shearing cycles (providing improved protection of surfaces upon shear) with respect to mimLUB alone, which weakly binds to surfaces and causes rapid wear as it is easily squeezed out of the shearing junction and also compared to FN films alone (Table 1). Collectively, these findings convey insights into the interactions our biomimetic polymer could potentially have with cartilage tissue through the FN layer present in the superficial zone.

Detailed structural imaging *via* AFM in air reveal that mimLUB molecules possess a worm-like structure (Figure 1), similar to what was reported for native LUB (Zappone et al., 2007). This worm-like structure likely provides flexibility to the polymer when grafted onto mica in high ionic strength solutions, facilitating exposure of its hydrated PEG side chains at the shearing interface to prevent (or at least delay) interdigitation between brushes anchored to opposite surfaces. When the sample is incubated at high concentrations, mimLUB forms a layer of evenly distributed agglomerates, as shown in Figure 2, providing full surface coverage. The adsorption method (rather than the spin-coated method) was chosen for SFA experiments because (i) it avoided the risk of the sample drying out and (ii) the ability of mimLUB to form a robust lubricating layer *via* adsorption from solution was of greater physiological/clinical relevance. Further, the presence of a thick underlying layer of FN enables distribution of stress throughout the film, which could delay the onset of wear. Our mimLUB polymer has a thiol moiety at one terminus to facilitate its binding to cartilage (that is to FN in our experiments) allowing us to compare it with the C-terminus of native LUB, as they are both implicated in the anchoring of the lubricating agent to the shearing surfaces (Jones et al., 2007). However, mimLUB lacks a moiety analogous to the N-terminus LUB possesses, utilized for self-aggregation (Jones et al., 2007). The mimLUB chains are, therefore, not capable of self-association into dimers to form loop-like conformations at the mica surface, as does native LUB (Andresen Eguiluz et al., 2015). Additionally, mimLUB’s contour length is approximately half of that of native LUB (Zappone et al., 2007). Collectively, these structural differences between native and mimetic LUB are likely responsible for distinctive binding and assembly mechanisms at the mica surface, which may also affect their tribological properties. Nevertheless, the simplicity of the design of mimLUB and the high control (pressure, temperature, shear velocity and sheared distance, surface roughness, film thickness, wear debris detection) achieved in our model system allowed us to unravel the role of FN in mediating the resistance to wear of mimLUB-coated surfaces during shear.

The fit of our normal forces data using the AdG model (Figure 3A) reveals additional interesting molecular details. Analysis of mimLUB alone indicates that the polymer chains are most likely lying down at the mica surface. Although the brush length extracted from the AdG fit is 20 nm, i.e., twice as large as the average molecular diameter, the raw (unfitted) data shows an onset of repulsion at circa 10 nm, which corresponds to one molecular diameter on each mica surface. In contrast, when mimLUB is incubated onto FN-coated mica, it self-assembles in a densely packed brush-like film, as suggested by a brush length of circa 260 nm (long-range interaction) and a grafting spacing of 14 nm. A brush-like length of 260 nm is significantly larger than the combined film thicknesses of mimLUB and FN alone. Such discrepancy could be attributed to the formation of loosely formed agglomerates of mimLUB (such as those revealed by AFM in Figure 2) and/or to conformational changes of either FN or mimLUB in the FN + mimLUB film compared to that of FN and mimLUB alone on bare mica. Under strong compression, the FN + mimLUB system exhibits a second, shorter-range brush behavior, which could be attributed to the ejection of unanchored agglomerates leaving behind a single layer of horizontally oriented mimLUB that is well-anchored to the underlying FN layer present on each mica surface and that remains trapped in the junction. Such a squeezing mechanism would also explain why the FN + mimLUB thickness under compression is approximately equal to the sum of FN and mimLUB film thicknesses.

Surprisingly, the presence of mimLUB in the shearing medium (Figure 6) provides only a modest improvement of surface protection against wear with respect to PBS (Figure 5) suggesting that surface-anchored mimLUB molecules, rather than free-floating mimLUB in the shearing medium, are responsible for enhanced lubrication and resistance to wear of the sheared surfaces. This finding strengthens the key role of FN in strongly anchoring mimLUB molecules to the surface so that they remain entrapped in the mica junction under high compressive pressures and during shear. The intermediate FN layer systematically leads to an extension of the wearless friction regime across both PBS (Figure 5B) and mimLUB (Figure 6B), with increased resistance to surface wear observed up to fivefold to sixfold more shearing cycles and up to higher loads. Despite higher measured friction coefficients (as compared to native LUB), the manifestation of wear only at high pressures indicates that mimLUB remains firmly anchored to mica not only under high compression/confinement but also when sheared across large distances (0.7 times the contact area) and over a wide range of velocities. Indeed, the contact pressures reached in our study without signs of wear are much higher than those reported for LUB on mica alone (Zappone et al., 2007) (0.4 MPa), and similar to those observed for LUB incubated on FN at low shearing velocities (≈3 MPa) (Andresen Eguiluz et al., 2015). It should be noted that the wear observed when shearing LUB on bare mica (Zappone et al., 2007) was not mica wear, but damage of the LUB film. In this work, however, neither FN + mimLUB film removal nor mica wear was observed at pressures up to 3.4 MPa in specific experimental conditions, which clearly indicates that FN + mimLUB offers better wear protection during shear than native LUB on bare mica and equivalent protection to native LUB on FN-coated mica.

It has been previously reported that strong anchoring of copolymers to shearing surfaces is a key prerequisite for efficient lubrication (Huang et al., 2001; Mueller et al., 2003; Chawla et al., 2009; Chen et al., 2009; Banquy et al., 2014a). Our findings confirm that a boundary lubricant needs to remain confined within surfaces to protect them efficiently, in this case, *via* FN. Using copolymer brushes, Raviv et al. (2003) showed that ultralow friction coefficients could be achieved in synthetic systems. However, the normal forces $F_{⊥}$ and shearing distances Δ*x* investigated in that report were significantly smaller (*F*_max_/*R* < 10 mN/m and Δ*x* = 0.7 µm, respectively) than those explored in our study (*F*_max_/*R* < 100 mN/m and Δ*x* = 35.0 µm). Here, we focused on exploring the tribology of *one* mimLUB configuration, but it is clear that backbone length (pAA molecular weight), side chain length (PEG molecular weight), and grafting distance between PEG side groups are all parameters that can be adjusted to optimize the tribological performance of the synthetic mimLUB. Additionally, the thiol moiety could easily be replaced by a peptide sequence, e.g., a hemopexin-like domain, to anchor to underlying substrates. The underlying layer of FN could also be replaced by other important structural proteins from cartilage, such as COL II or HA, which may affect the anchoring of mimLUB to the surface and consequently its lubrication. Similar systems have recently been investigated by other groups (Faivre et al., 2017). Current work utilizing other copolymer configurations and multiple-binding domains that better mimic LUB’s tethering to the cartilage tissue surface is in progress in our group.

Finally, we should point out that our model system utilizes stiff and non-porous confining surfaces whereas cartilage is compliant and highly porous. Indeed, pores contribute to the ability of cartilage to carry high loads by distributing the loading pressure through the synovial fluid contained (and circulating) within them, a mechanism known as poroelasticity. Such load distribution effectively reduces friction coefficients during locomotion (Hodge et al., 1986). The use of mica as a model substrate was chosen because it reduces the complexity of the system. As mica is atomically smooth and non-porous, it provides a well-defined model surface ensuring us that all tribological events observed can be attributed to changes in the interfacial polymer, i.e., to phenomena occurring at the junction between surfaces rather than within surfaces.

## Conclusions and Future Work

We characterized a synthetic mimLUB and the role of FN in mediating the friction and wear between mimLUB-coated mica surfaces upon shear, at the molecular scale. We found that the presence of FN serves as a key contributor to link mimLUB to either cartilage or implants for enhancing wear protection during prolonged shear up to contact pressures of 3.4 MPa. This effect was attributed to FN-mediated strong anchoring and consequent robust entrapment of mimLUB within the mica junction, which enables the formation and retention of a dense repulsive brush preventing interdigitation and removal of mimLUB chains when surfaces are sheared, even under high pressures. Furthermore, we demonstrated that surface-bound, rather than free-floating mimLUB is central to providing good wear protection. These findings provide insights into the (synergistic) lubrication mechanisms of multicomponent systems and suggest that our proposed synthetic mimetic could be a potential effective alternative to natural LUB for the treatment of damaged cartilage surfaces through the FN layer present in its superficial zone.

Further functionalization of mimLUB with multiple-binding domains that would improve its tethering properties to cartilage tissues and prosthetic implants, and the design of other mimetics configurations that would yield both enhanced protection and lower friction are currently being explored in our group.

## Author Contributions

RAE, SC, and DG wrote the manuscript. RAE, LB, DP, and DG designed experiments. MT synthesized mimLUB. RAE, SC, MT, CB, and NP analyzed data. RAE, SC, MT, MS, LB, DP, and DG interpreted and discussed results. DG supervised the project.

## Conflict of Interest Statement

The authors declare that the research was conducted in the absence of any commercial or financial relationships that could be construed as a potential conflict of interest.
